# The diagnosis and management of NK/T-cell lymphomas

**DOI:** 10.1186/s13045-017-0452-9

**Published:** 2017-04-14

**Authors:** Eric Tse, Yok-Lam Kwong

**Affiliations:** Department of Medicine, Professorial Block, Queen Mary Hospital, Pokfulam Road, Hong Kong, China

**Keywords:** NK/T-cell lymphoma, Extranodal, Nasal, Non-nasal, EBV DNA quantification, L-asparaginase, PD1, Immunotherapy, Prognostication

## Abstract

Extranodal natural killer (NK)/T-cell lymphoma is an aggressive malignancy of putative NK-cell origin, with a minority deriving from the T-cell lineage. Pathologically, the malignancy occurs in two forms, extranodal NK/T-cell lymphoma, nasal type; and aggressive NK-cell leukaemia. Lymphoma occur most commonly (80%) in the nose and upper aerodigestive tract, less commonly (20%) in non-nasal areas (skin, gastrointestinal tract, testis, salivary gland), and rarely as disseminated disease with a leukemic phase. Genetic analysis showed mutations of genes involved in the JAK/STAT pathway, RNA assembly, epigenetic regulation, and tumor suppression. In initial clinical evaluation, positron emission tomography computed tomography, and quantification of plasma EBV DNA are mandatory as they are useful for response monitoring and prognostication. In stage I/II diseases, combined chemotherapy and radiotherapy (sequentially or concurrently) is the best approach. Conventional anthracycline-containing regimens are ineffective and should be replaced by non-anthracycline-containing regimens, preferably including L-asparaginase. Radiotherapy alone is associated with high systemic relapse rates and should be avoided. In stage III/IV diseases, non-anthracycline-regimens-containing L-asparaginase are the standard. In relapsed/refractory cases, blockade of the programmed death protein 1 has recently shown promising results with high response rates. In the era of effective non-anthracycline-containing regimens, autologous haematopoietic stem cell transplantation (HSCT) has not been shown to be beneficial. However, allogeneic HSCT may be considered for high-risk or advanced-stage patients in remission or relapsed/refractory patients responding to salvage therapy. Prognostic models taking into account presentation, interim, and end-of-treatment parameters are useful in triaging patients to different treatment strategies.

## Background

Natural killer (NK) cells constitute the third lymphoid lineage, in addition to T-cell and B-cell lineages. Developmentally, NK-cells and T-cells share a related ontogeny, arising from a common lymphoid progenitor [1]. Expression of distinct transcription factors leads to different lineage commitment, with expression of ID2 and E4BP4 resulting in development into the NK-cell lineage, and expression of the NOTCH and RUNX members resulting in T-cell lineage development [1]. Conventionally, NK-cells are typically regarded to be part of the innate immune system, implying that they are able to proliferate rapidly in response to antigens without prior sensitization, and that their lifespans are short without a long-lived memory population. In contrast, T-cells are educated in the thymus with exposure to different spectra of antigens and develop into memory T-cells after antigen-driven proliferation; which are the standard properties of the adaptive immune system [1]. Interestingly, recent observations have shown that NK-cells may also acquire immunological memory [2], and that they can persist as long-lived cells [1]. Hence, the distinction of NK-cells as belonging to the innate immune system is blurred. However, regarding NK-cells as part of the innate immune system is still useful. These conceptual aspects include the residence of NK-cells in non-nodal sites, which is reflected pathologically by localization of NK-cell malignancies in extranodal sites. Moreover, NK-cells lack recombination activating gene enzymes for receptor gene rearrangement, so that unique antigen recognition receptors are absent. Hence, in neoplastic NK-cells, a clonal molecular marker due to receptor gene rearrangement is not present [3].

NK-cells develop predominantly in non-nodal sites, including the liver and the bone marrow [4]. They are cytolytic cells expressing cytotoxic molecules including granzyme B and perforin. Immunophenotypically, NK-cells express CD2, cytoplasmic CD3 epsilon (ε), but not surface CD3, CD16, CD56, CD94, and killer-cell immunoglobulin-like receptors (KIRs) [3]. KIRs are important molecules that bind HLA class I molecules as their ligands. Ligation of KIRs with HLA class I molecules results in transduction of inhibitory signals, which serves to protect normal cells expressing HLA class I molecules from NK-cell-mediated cytotoxicity. However, virally infected and neoplastic cells downregulate HLA class I molecules, rendering them susceptible to targeting by NK-cells [3].

### Malignancies of putative NK-cell origin

It had been known for a long time that rare destructive midline facial lesions might develop in some patients, which led relentlessly to death. These lesions were referred to as lethal midline granuloma [5]. With advances in pathology, they were recognized to be neoplasms of lymphoid origin. The neoplastic infiltrate comprised a polymorphic population of atypical lymphoid cells, inflammatory cells, and eosinophils, which was quite different from conventional lymphomas where neoplastic cells were more homogeneous. Hence, the notation of polymorphic reticulosis was adopted [6]. The development of immunohistochemistry further improved the classification of this lymphoma. Initially, staining of formalin-fixed paraffin-embedded specimens with polyclonal antibodies showed that the T-cell antigen CD3 was expressed on neoplastic cells. Furthermore, lymphoma cells showed a propensity to invade and destroy blood vessels. Hence, they were originally classified as angiocentric T-cell lymphomas in the REAL classification of lymphoid malignancies [7].

Once monoclonal antibodies were developed to work on frozen or cryostat sections, these angiocentric T-cell lymphomas were found not to express surface CD3. The CD3 detected previously with polyclonal antibodies on paraffin-embedded sections represents detection of the cytoplasmic ε chain of CD3 [8], a characteristic of NK-cells instead. Finally, molecular analysis of the T-cell receptor (TCR) gene shows a germline configuration in the majority of cases [6]. Therefore, these lymphomas are in fact of putative NK-cell origin. Interestingly, as more cases were examined, some of these lymphomas appeared to be of bona fide T-cell origin, with clonal rearrangement of the *TCR* gene [9, 10]. Nevertheless, the putative cellular origins of these lymphomas have no impact on the clinical characteristics and response to treatment [9, 10]. In the latest 2016 World Health Organization (WHO) classification of lymphoid malignancies, these lymphomas are referred to as extranodal NK/T-cell lymphoma, to reflect putative cellular origins from both NK-cells and T-cells [11].

### Pathological features of NK/T-cell lymphomas

**Fig. 1:**
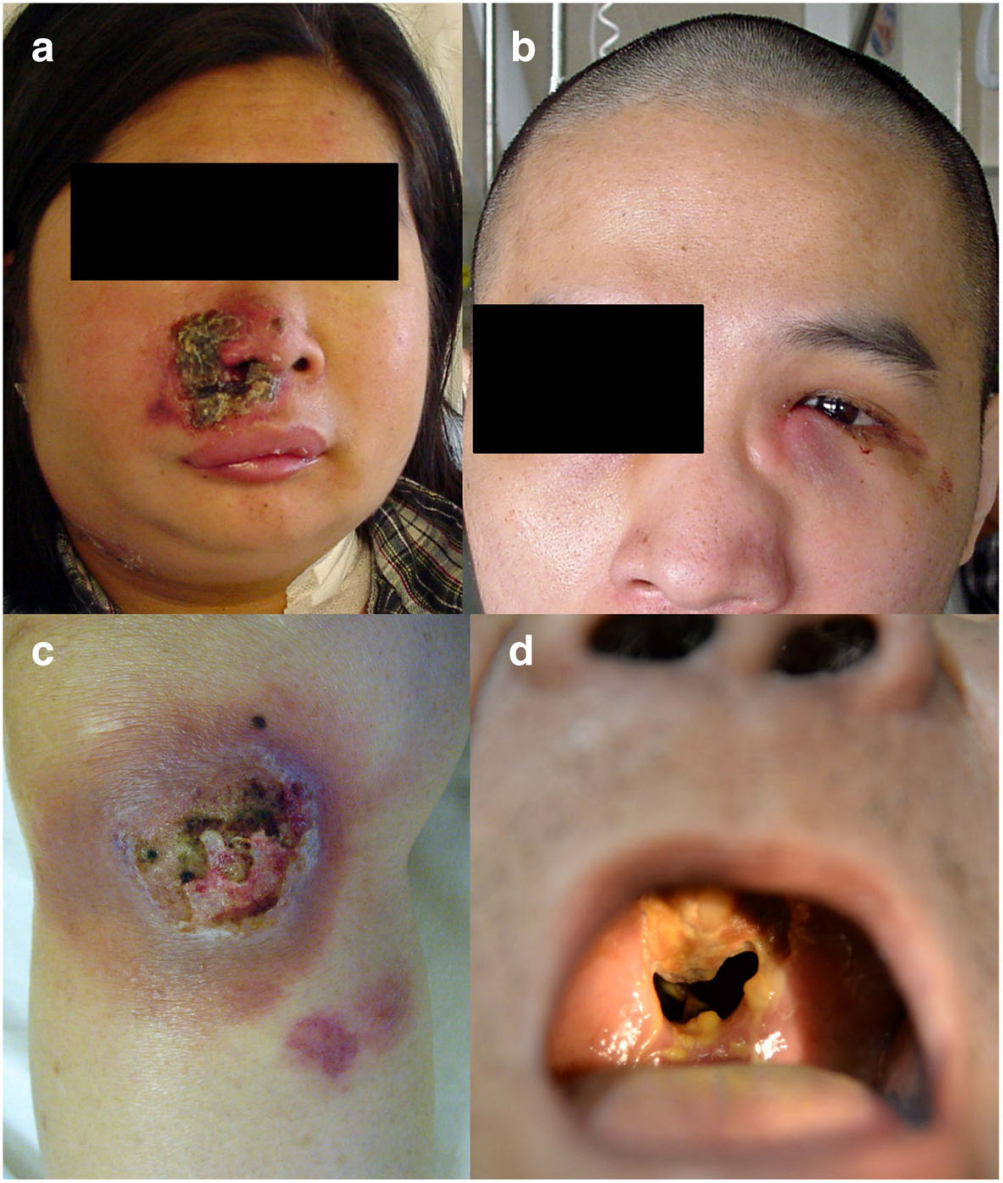
Clinical features of NK/T-cell lymphomas. **a** Nasal lesion that has ulcerated into the face. **b** Nasal lesion with extension to the orbit. **c** Cutaneous lesion in the knee that has ulcerated. Note the two adjacent lesions in their early stages. **d** Perforation of the hard palate, leading to a communication between the oral and nasal cavities

NK/T-cell lymphomas develop almost exclusively in non-nodal sites. About 80% of cases occur in the nose, nasopharynx, oropharynx, the Waldeyer’s ring, and parts of the upper aerodigestive tract (Figs. 1a, b). Collectively, these lymphomas are referred to as nasal NK/T-cell lymphomas [12]. About 20% of these lymphomas occur in non-nasal sites, including the skin (Fig. 1c), testis, gastrointestinal tract, muscle, and salivary glands and are referred to as non-nasal NK/T-cell lymphomas [12]. Rarely, the lymphoma can be disseminated on presentation, with infiltration of the liver, spleen, skin, lymph nodes, and bone marrow. Involvement of the peripheral blood is frequently found. These cases are referred to as aggressive NK-cell leukemia/lymphoma [11, 12].

Histologically, neoplastic cells are similar irrespective of their anatomical localization. Neoplastic infiltrates often exhibit angiocentricity and angiodestruction, leading to zonal necrosis. Lymphoma cells express the typical immunophenotype of CD2+, surface CD3–, cytoplasmic CD3ε+, CD56+, and cytotoxic molecules (perforin, granzyme B, T-cell intracellular antigen 1, TIA1)+. Cytologically, neoplastic cells are large granular lymphocytes. In disseminated cases, active haemophagocytosis may be found in the liver, spleen, and bone marrow, leading to impaired liver function, hyperferritinaemia, and pancytopenia [13]. A defining feature of all types of NK/T-cell lymphoma is the invariable infection of lymphoma cells with Epstein-Barr virus (EBV), which exists in an episomal form not integrated into the host genome [5]. Furthermore, assessment by terminal repeat sequences shows EBV in lymphoma cells to be clonal, implying that the infection has occurred either before or at the time of lymphomagenesis. Therefore, EBV infection very likely plays an important part in NK-cell lymphomagenesis. Accordingly, demonstration of EBV infection is a pre-requisite for the diagnosis of NK/T-cell lymphoma [11]. In the routine laboratory, EBV is usually detected by in situ hybridization for EBV early RNA (EBER). Absence of EBV excludes the diagnosis of NK/T-cell lymphoma. However, EBV is required, but not adequate for the diagnosis. Either CD56 or cytotoxic molecules (granzyme B, perforin, TIA1) must be present. In the absence of both, the diagnosis becomes EBV-positive peripheral T-cell lymphoma, not otherwise specified [11].

### Molecular pathogenesis of NK/T-cell lymphomas

The first genetic aberration found in NK/T-cell lymphomas was deletion of chromosome 6q (6q–) on karyotypic analysis [14]. Results of comparative genomic hybridization [15] and loss of heterozygosity analyses [16] also showed that 6q– was common. Later investigations assigned a number of putative tumor suppressor genes to this segment of chromosome 6q, which included *HACE1* [17], *PRMD1* [18], *FOXO3* [19], and *PTPRK* [20]. The use of gene expression profiling (GEP) had revealed other findings. NK/T-cell lymphoma and peripheral T-cell lymphoma (PTCL) of γδ subtype were shown to have similar patterns of GEP, consistent with their development relationship [21]. Furthermore, oncogenic mechanisms involved in NK/T-cell lymphomas, including activation of the JAK/STAT pathway, and over-expression of NK-κB and aurora kinase A, had also been uncovered by GEP [22, 23].

With the advent of next generation sequencing, the mutational landscape of NK/T-cell lymphoma becomes better defined (Table 1). Exome sequencing showed *JAK3* mutations, which led to constitutive activation of the JAK/STAT pathway in about 35% of cases [24]. Later studies failed to confirm a high incidence of *JAK3* mutation, but the importance of the JAK/STAT pathway was shown by activating mutations of *STAT3* and *STAT5B* in a sizeable number of cases [25, 26]. Results from these studies imply that the JAK/STAT pathway may be targetable therapeutically.

**Table 1:** Somatic gene mutations identified in NK/T-cell lymphoma

Gene	Frequency	Reference
DDX3X	20%	27
JAK3	5–35%	24, 26
STAT3	6–27%	25–27
STAT5B	2–6%	25, 27
BCOR	21%	26
MLL2	7–18%	26, 27
TP53	12–13%	26, 27
KRAS	6%	26
ASXL3	4%	27
ARID1A	6%	27
EP300	4%	27

In another study involving 105 cases, *DDX3X* mutations were identified in about 20% of cases [27]. DDX3X is an RNA helicase involved in RNA translation initiation and assembly in the ribosome and spliceosome. DDX3X mutants showed decreased RNA-unwinding, resulting in loss of cell-cycle suppression and transcriptional activation of the NF-κB and MAPK pathways. Other mutations included the tumor suppressor gene *TP53*, found in about 13% of cases [27], and *MLL*, *ASXL1*, *ARID1A*, and *EP300*, which are involved in epigenetic pathways, in 4–18% of cases [26, 27].

Epigenetic inactivation of putative tumor suppressor genes had also been found in NK/T-cell lymphomas [28, 29]. The potential therapeutic significance of these changes has not been explored.

Data on aggressive NK-cell leukaemia are scarce, owing to the rarity of the disease. In one study of 35 patients, mutation of *STAT5B* was identified in one of 5 cases, whereas promoter methylation of *HACE1*, which encodes an E3 ubiquitin ligase and is a potential tumor suppressor gene, was found in 3 of 4 cases [30]. Interestingly, although the predominant majority of aggressive NK-cell leukemia is EBV-positive, a series of seven cases of EBV-negative aggressive NK-cell leukaemia had been reported recently [31]. In two of these cases, *STAT3* mutations were found. The pathogenetic mechanisms of EBV-negative NK-cell leukaemias remain to be defined.

### Clinical features of NK/T-cell lymphoma

**Fig. 2:**
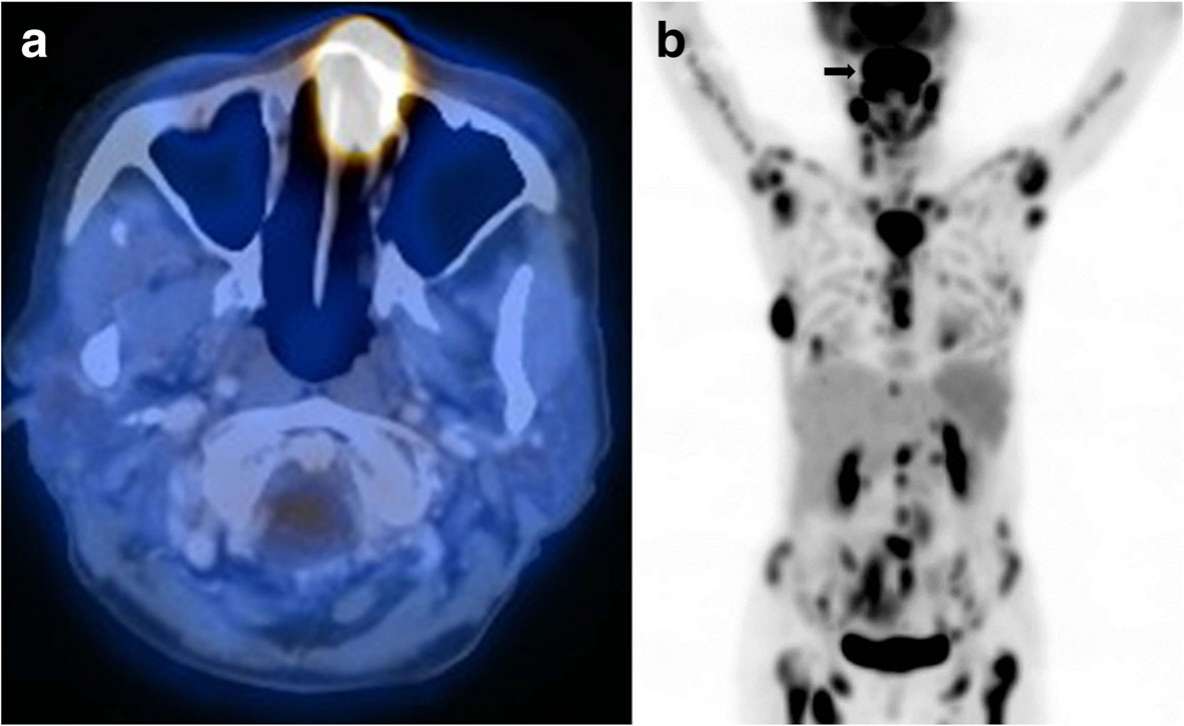
Positron emission tomography computed tomography of NK/T-cell lymphomas. **a** Nasal lesion that shows avidity for 18F-fluorodeoxyglucose. **b** Disseminated disease. Note the large nasal tumor (*arrow*) and multiple hypermetabolic lesions in other anatomical sites

Nasal NK/T-cell lymphomas show a geographic predilection for Asian and South American populations, and are uncommon in other countries [6]. Its incidences in Southeast Asian and Central/South American countries were 5.2 and 3%, respectively, as compared with just 0.3% in North American and European countries [32]. The lymphoma is locally invasive. Because of angiodestruction, the lymphoma destroys midline facial structures, often manifesting as hard palate perforation (Fig. 1d) [33]. Other facial structures may also be involved, including the orbit, salivary glands, and paranasal sinuses. In non-nasal NK/T-cell lymphoma, commonly involved primary sites include the skin [34], gastrointestinal tract [35, 36], and testis [37]. Occasional involvement of rare sites including the muscle [38] and uterus [39] had also been reported. NK/T-cell lymphomas are predominantly extranodal, and primary nodal presentation is exceptionally rare [40]. It must be noted that when NK/T-cell lymphomas appear to present primarily in non-nasal sites, F18 florodeoxyglucose (FDG) positron emission tomography/computed tomography (PET/CT) is needed to exclude an occult nasal primary (practically all NK/T-cell lymphomas are FDG-avid [41, 42]) (Fig. 2a, b). If nasal involvement is found, these “non-nasal” cases should be regarded as nasal lymphomas with systemic spread [43]. Hence, a strict definition of non-nasal NK/T-cell lymphoma requires exclusion of a nasal primary on PET/CT.

Aggressive NK-cell leukaemia/lymphoma is the rarest presentation of an NK/T-cell malignancy, currently categorized by the WHO classification as aggressive NK-cell leukaemia [11]. Patients present with fever, lymphadenopathy, rashes, hepatosplenomegaly, hyperferritinaemia, and pancytopenia. Active haemophagocytosis is found in the marrow and other organs, which accounts for many of the disease manifestations [44]. Clinical course is relentlessly downhill, with survival often measured only in weeks to months.

### Quantification of circulating EBV DNA

NK/T-cell lymphoma is universally infected by EBV. When lymphoma cells undergo apoptosis, fragments of EBV DNA of <500 base-pairs are released into the blood. Quantification of circulating EBV DNA provides an accurate surrogate biomarker of lymphoma load [45–47]. Whole blood had been used in some studies for EBV DNA quantification in NK/T-cell lymphomas. Whole blood, however, is not the suitable material. Firstly, EBV DNA has to be quantified per unit of total DNA, which varies according to the leucocyte count. Secondly, circulating long-lived memory B-cells are most often infected by EBV, and their unpredictable numbers in whole blood introduce errors that cannot be controlled. Thirdly, leukocyte DNA may cause technical problems with the quantitative polymerase chain reaction (Q-PCR). Therefore, plasma is the better starting materials. In a recent study of EBV-related lymphoid malignancies other than NK/T-cell lymphoma, plasma has been confirmed to be superior to whole blood for EBV DNA quantification [47].

Plasma EBV DNA quantification at diagnosis gives an accurate measurement of the lymphoma load [45]. It also provides a dynamic measurement of how the lymphoma responds to treatment [46]. Finally, at the end of treatment, EBV DNA gives an estimate of minimal residual disease, which is of prognostic significance [48].

### Differential diagnoses of NK/T-cell lymphomas

Several conditions simulate NK/T-cell lymphomas. Plasmacytoid dendritic cell neoplasms, formerly erroneously classified as blastoid NK-cell lymphomas, are localized mainly to the skin but may also involve the bone marrow [49]. It can easily be distinguished from cutaneous NK/T-cell lymphomas by the absence of CD3, cytotoxic molecules, and EBV infection. NK-cell lymphomatoid gastropathy is a self-limiting non-neoplastic proliferation of NK-cells [50]. It is localized to the stomach, but cases involving the small and large bowels have also been described (NK-cell enteropathy) [51]. Histologically and immunophenotypically, lymphomatoid gastropathy/NK-cell enteropathy and NK/T-cell lymphoma share many similarities. The key difference is that EBV is absent in lymphomatoid gastropathy/NK-cell enteropathy. Lesions may self-regress, although occasional cases may relapse. Metastasis to other organs does not occur. Chronic lymphoproliferative disorder of NK-cells is a rare and heterogeneous disorder. It is currently unclear whether the disorder is reactive or neoplastic, owing to the lack of a clonal marker for NK-cells. It is not associated with EBV infection and can be easily distinguished from aggressive NK-cell leukaemia by its indolent and non-progressive nature. Most patients are asymptomatic, and treatment is not required.

### Initial evaluation of newly diagnosed patients

In the initial evaluation of newly diagnosed patients, history of conditions that predispose to NK/T-cell lymphoma should be sought. Chronic active EBV infection (CAEBV) is an uncommon condition, characterized by a prolonged period of illness (>6 months) with fever, lymphadenopathy, splenomegaly, and impaired liver function. Patients have elevated EBV antibodies, high levels of circulating EBV DNA, and organ infiltration by EBER+ T-cells or NK-cells [52, 53]. Progression of CAEBV may result in systemic EBV+ T-cell lymphoproliferative diseases, or an NK-cell malignancy, almost always presenting as aggressive NK-cell leukaemia/lymphoma [54, 55]. Mosquito-bite hypersensitivity is a rare condition, which can exist for a long time, or as part of the manifestation of CAEBV [52]. Patients develop blistering and sometimes ulcerating lesions after insect bites, which may be associated with systemic symptoms of fever and lymphadenopathy. Lesions heal with scarring, but when NK/T-cell lymphoma (occasionally T-cell lymphoma) has finally developed, the ulcer does not heal, leading to the diagnosis of a malignancy.

Laboratory evaluation includes blood count and serum biochemistry. Trephine biopsy is needed to define if marrow infiltration has occurred. Morphologic examination of the trephine biopsy should be supplemented with in situ hybridization for EBER. The presence of EBER+ atypical cells indicates marrow infiltration [6]. Plasma EBV DNA quantification gives information on the starting tumor load, and provides a biomarker to gauge treatment response.

Conventional imaging by CT and magnetic resonance imaging (MRI) gives important information on local tumor extent, with CT more sensitive for bony lesions and MRI better for soft tissue lesions. While CT and MRI are good imaging modalities for nasal lesions, they are inadequate for detecting small or subclinical systemic metastases. For non-nasal primaries, CT and MRI are not useful for staging because occult involvement in the nasal and other anatomical sites cannot be detected. Hence, in previously reported clinical studies of NK/T-cell lymphomas of the non-nasal type where only CT had been performed, data are no longer considered to be reliable.

PET/CT is currently regarded as the standard imaging modality for NK/T-cell lymphoma [41, 42]. Although it is an aggressive lymphoma, the maximum standard uptake value of lesions is typically less than that of other aggressive B-cell lymphomas [41]. It is important for newly diagnosed patients to undergo PET/CT scan, not only for accurate staging, but also because interim and end-of-treatment scan results may be prognostic [56].

### General principles of management of NK/T-cell lymphomas

Before the lineage and pathology of NK/T-cell lymphomas was clearly delineated, patients were treated with anthracycline-containing (CHOP, cyclophosphamide, adriamycin, vincristine, prednisolone, or CHOP-like) regimens as for other aggressive lymphomas [55]. However, it soon became clear that such regimens worked poorly. This has been attributed to high expression levels of the multidrug resistance (MDR) P-glycoprotein on NK lymphoma cells, rendering drugs exported by P-glycoprotein (such as cyclophosphamide and adriamycin) ineffective [57]. Consequently, CHOP or CHOP-like regimens have now been abandoned in the treatment of NK/T-cell lymphoma patients and have been replaced by an array of more effective non-anthracycline-containing regimens [12].

### Management of stage I/II NK/T-cell lymphoma

Similar to other lymphomas, the treatment modalities for stage I/II NK/T-cell lymphoma include radiotherapy, chemotherapy, or their combinations (Table 2). The optimal approach remains undefined, as large prospective clinical studies comparing each treatment modality has not been conducted [58].

**Table 2:** Selected clinical studies on the treatment of stage I/II NK/T-cell lymphoma

Study (reference)	No.	Treatment	ORR	CR	OS	PFS
Retrospective [59]	253	RT (50 Gy)	NR	NR	5-year: 70%	5-year: 65%
Phase II [61, 65]	150	CCRT (50 Gy) + 2/3 DeVIC	89%	82%	5-year: 72%	5-year: 61%
Phase II [63]	30	CCRT (40 Gy) + VIPD	83%	80%	3-year: 86%	3-year: 85%
Phase II [64]	30	CCRT (40 Gy) + VIDL	90%	87%	5-year: 73%	5-year: 60%
Phase II [69, 70]	26	LVP + sandwiched RT (56 Gy)	89%	81%	5-year: 64%	5-year: 64%
Phase II [70, 71]	27	GELOX + sandwiched RT (56 Gy)	96%	74%	5-year: 85%	5-year: 74%
Phase II [73]	33	DICE-L + RT (45 Gy)	91%	100%	5-year: 89%	5-year: 82%
Retrospective [68]	29	SMILE + sandwiched RT (50 Gy)	90%	69%	NR	NR

*No.* number of patients, *ORR* overall response rate, *CR* complete response rate, *OS* overall survival, *PFS* progression-free survival, *NR* not reported, *RT* radiotherapy, *CCRT* concurrent chemoradiotherapy, *DeVIC* dexamethasone, etoposide, ifosfamide, carboplatin, *VIDP* etoposide, ifosfamide, cisplatin, dexamethasone, *VIDL* etoposide, ifosfamide, dexamethasone, L-asparaginase, *LVP* L-asparaginase, vincristine, prednisoloen, *GELOX* gemcitabine, L-asparaginase, oxaliplatin, *DICE-L* dexamethasone, ifosfamide, cisplatinum, etoposide, L-asparaginase, *SMILE* dexamethasone, methotrexate, ifosfamide, L-asparaginase, etoposide

### Radiotherapy

NK/T-cell lymphoma cells are radiosensitive, and radiotherapy used to be employed in the first-line treatment of stage I/II disease. Despite an apparent good initial overall response rate (ORR) and complete response (CR) rate, the use of radiotherapy alone is associated with an incidence of systemic relapse that is alarmingly high for early-stage patients [58]. In a retrospective analysis of early-stage nasal NK/T-cell lymphoma with low risk features (defined in the study as age <60 years, Eastern Cooperative Oncology Group performance score <2, stage I disease, normal lactate dehydrogenase, and no primary tumor invasion into surrounding tissues), the 5-year progression-free survival (PFS) and overall survival (OS) were 79.2 and 88.8% for patients receiving radiotherapy alone [59]. A relapse rate of 18.8% was reported, which in such good-risk patients was unacceptably high. With the addition of ineffective anthracycline-containing chemotherapy to radiotherapy, the outcome was expectedly not improved (PFS: 81.6%; OS: 86.9%). The use of anthracycline-containing regimens followed by radiotherapy gave even poorer results (PFS: 71.5%; OS: 86.3%). In a more recent study where radiotherapy was given with or without chemotherapy (80% of which was anthracycline-containing) for apparently unselected patients, the results were worse [60], with a 5-year PFS of merely 61%, and a 5-year OS of 70%, even when high doses of radiotherapy (at least 50 Gy) were used. Hence, for stage I/II patients in whom chemotherapy (non-anthracycline-containing) is feasible, radiotherapy should not be employed as the sole initial treatment, whatever the risk categories. When radiotherapy is combined with chemotherapy, non-anthracycline-containing regimens instead of CHOP or CHOP-like regimens should be used. However, radiotherapy alone for stage I/II disease might be considered appropriate in centers where chemotherapy cannot be safely given, or in elderly individuals with poor performance, for whom chemotherapy is considered unsafe.

### Concurrent chemoradiotherapy

Two prospective phase I/II trials evaluated the efficacy of concurrent radiotherapy given with platinum-containing regimens (concurrent chemoradiotherapy, CCRT) for the treatment of localized stage I/II NK/T-cell lymphoma, based on the observation that platinum sensitized solid tumors to radiotherapy. In the first study, 33 patients were treated with CCRT (50 Gy) and 3 cycles of two-third DeVIC (dexamethasone, etoposide, ifosfamide and carboplatin) [61, 62]. The ORR and CR rate were 78 and 75%. Grade 3/4 neutropenia was observed in 93% of patients and grade 3 radiation-related mucositis in 30%. With a median follow-up of 68 months, the 5-year OS and PFS were 73 and 67% [62]. In the second study, 30 patients with stage I/II NK/T-cell lymphoma were treated with CCRT (40 Gy + cisplatinum. 30 mg/m^2^ weekly), followed by 3 cycles of VIPD (etoposide, ifosfamide, cisplatin, and dexamethasone) [63]. The ORR and CR rate were 83.3 and 80%. Systemic relapse was found in 6.6% of patients. Grade 1/2 mucositis was observed in 11% of patients and grade 3/4 leucopenia in 14%. The 3-year OS and PFS were 86.3 and 85.2%.

In an effort to improve outcome, CCRT followed by an L-asparaginase-containing regimen (VIDL, etoposide, ifosfamide, dexamethasone, L-asparaginase) was tested in 30 patients with stage I/II NK/T-cell lymphoma [64]. The ORR and CR rate were 90 and 87%. Grade 3/4 mucositis was observed in 16.6% of patients and grade 3/4 leucopenia in 80%. With a median follow-up of 44 months, the 5-year OS and PFS were 60 and 73%. Results were not significantly different from those of other CCRT trials.

Another study examined the use of CCRT in everyday clinical practice. In 150 patients with stage I/II NK/T-cell lymphoma treated with CCRT and DeVIC, the ORR and CR rate was 89 and 82% [65]. Grade 3/4 febrile neutropenia was observed in 17% of patients and grade 3/4 mucositis in 38%. With a median follow-up of 5.6 years, the 5-year PFS and OS were 61 and 72% [65]. The results were comparable with those observed in previous clinical trials [61, 62].

These studies showed that CCRT resulted in similar outcome inside and outside clinical trials. However, CCRT has important limitations. Radiotherapy is not immediately available in many centers. Furthermore, radiotherapy administered with chemotherapy causes substantial mucosal toxicity, and tolerability is poor in patients who have more locally advanced tumors. Consequently, CCRT is not practiced routinely outside Japan and South Korea.

### Sequential chemotherapy and radiotherapy

With the advent of L-asparaginase, the outlook of NK/T-cell lymphomas has been entirely changed. L-asparaginase was demonstrated to have excellent in vitro anti-NK-cell lymphoma activity [66]. In the first international phase II study of the L-asparaginase-containing regimen SMILE (dexamethasone, methotrexate, ifosfamide, L-asparaginase and etoposide)[67], an ORR of 79% was observed in relapsed and refractory patients with advanced-stage disease. Based on these encouraging results, another international study examined the use of SMILE with sandwiched radiotherapy in 29 stage I/II patients [68]. After the initial 2–3 cycles of SMILE, the ORR and CR rate were 86.2 and 69%. On completion of sandwiched radiotherapy (50 Gy) and the subsequent 3–4 cycles of SMILE, the ORR was increased to 89.7%, and the CR rate remained unchanged; suggesting that the response to SMILE was rapid and was seen even after the first 2 cycles. Furthermore, the response was durable, with 90% of patients remaining in CR during follow-up. Grade 3/4 neutropenia was observed in 60.5% of patients. Treatment-related mortality was 7%, occurring mainly in the initial phase of the study when vigorous granulocyte colony stimulating factor (G-CSF) support was not mandated. With institution of active G-CSF support, no treatment-related mortality was later observed.

Other L-asparaginase-containing regimens in combination with radiotherapy have since been examined. A phase II study of LVP (L-asparaginase, vincristine, and prednisolone) with sandwiched radiotherapy was conducted in 26 patients with stage I/II NK/T-cell lymphoma [69]. A CR rate of 80.8% was reported. Grade 3 leucopenia was observed in 2.7% of patients and grade 3 radiation-related mucositis in 23.1%. With a median follow-up of 67 months, the 5-year OS and PFS were both 64% [70]. The efficacy and safety of another L-asparaginase-containing regimen GELOX (gemcitabine, L-asparaginase, and oxaloplatin) together with sandwich radiotherapy was examined in 27 patients with stage I/II NK/T-cell lymphoma [71]. The ORR and CR rate were 96 and 74%. Grade 3/4 leucopenia developed in 33.3% of patients, and grade 3 radiation-related mucositis in 15%. With a median follow-up of 63.2 months, the 5-year OS and PFS were 85 and 74% [72]. In a retrospective analysis, 33 patients with stage I/II disease were treated with 4 cycles of DICE-L-asp (dexamethasone, ifosfamide, cisplatinum, etoposide, and L-asparaginase), followed by radiotherapy as consolidation [73]. The ORR was 100%, with CR achieved in 90.9%. Local and systemic relapse rates were both 3%. Grade 3/4 leucopenia developed in 75.8% of patients and grade 3/4 radiation mucositis in 6.1%. With a median follow-up of 60 months, the 5-year OS and PFS were 89.2 and 82.9%.

These results showed that chemotherapy followed by radiotherapy gave very good results. Although this approach has not been directly compared with CCRT, the available data suggest that there are no significant differences in outcome for patients treated by both modalities. Therefore, either CCRT or sequential chemotherapy and radiotherapy can be accepted as first-line treatment for stage I/II NK/T-cell lymphoma patients, provided that effective non-anthracycline regimens particularly those containing L-asparaginase are used.

### Newly diagnosed advanced-stage (III/IV) NK/T-cell lymphoma

Non-anthracycline-containing regimens are currently the standard (Table 3). In a phase II study, twenty newly diagnosed stage-IV patients were treated with SMILE [67]. After 2 cycles of SMILE, the ORR and CR rate were 80 and 40%. Grade 3/4 neutropenia occurred in all patients and treatment-related mortality was 10%. Other significant non-haematological toxicities included transaminitis and renal function impairment. With a median follow-up of 24 months, the 1-year OS and PFS were both 45%. In another retrospective analysis of 26 patients with newly diagnosed stage IV NK/T-cell lymphoma, 14 (53.8%) patients achieved CR after SMILE chemotherapy [68]. Grade 3/4 neutropenia occurred in 60% of patients. The 5-year OS was 47.4%, and the 4-year disease-free survival (DFS) was 60% [68].

**Table 3:** Selected clinical studies on advanced-stage and relapsed/refractory NK/T-cell lymphomas

Study (reference)	Disease	No.	Treatment	ORR	CR	OS	PFS/DFS
Phase II [67]	Newly diagnosed stage IV, relapsed/refractory	38	SMILE	79%	45%	1-year: 55%	1-year: 53%
Retrospective [68]	Newly diagnosed stage IV	26	SMILE	NR	54%	5-year: 47%	4-year: 60%
Phase III [74]	Newly diagnosed, advanced stage	21	DDCP	95%	71%	1-year: 90%	1-year: 86%
		21	SMILE	67%	29%	1-year: 57%	1-year: 38%
Phase II [75]	Newly diagnosed, advanced stage	22	IMP L-asp	90%	65%	1-year: 76%	1-year: 43%
Phase II [76]	Relapsed/refractory	19	AspaMetDex	78%	61%	2-year: 40%	2-year: 40%
Retrospective [68]	Relapsed/refractory	44	SMILE	77%	66%	5-year: 52%	4-year: 68%
Retrospective [77]	Relapsed/refractory	13	MEDA	77%	61%	1-year: 69%	1-year: 62%
Retrospective [84]	Relapsed/refractory	7	Pembrolizumab	100%	71%	NR	NR

*No.* number of patients, *ORR* overall response rate, *CR* complete response, *OS* overall survival, *PFS* progression-free survival, *DFS* disease-free survival, *SMILE* dexamethasone, methotrexate, ifosfamide, L-asparaginase, etoposide, *DDCP* dexamethasone, gemcitabine, cisplatinum, pegasparaginase, *IMP L-asp* ifosfamide, methotrexate, etoposide, prednisolone, L-asparaginase, *AspaMetDex* L-asparaginase, methotrexate, dexamethasone, *MEDA* methotrexate, etoposide, dexamethasone, L-asparaginase

Another L-asparaginase-containing regimen DDGP (dexamethasone, gemcitabine, cisplatinum, and pegaspargase) was compared with SMILE in a phase III randomized trial for untreated advanced-stage NK/T-cell lymphoma [74]. Twenty-one patients received DDGP, achieving ORR and CR rate of 95 and 71%. With a median follow-up of 14 months, the 1-year OS and PFS were 90 and 86%. For the 21 patients receiving SMILE, the ORR and CR rate were 67 and 29%, and the 1-year OS and PFS were 57 and 38%. The results of SMILE in this study were significantly inferior to those reported in previous well-conducted studies [67, 68], without any clear reasons. Therefore, the apparent advantage of DDGP over SMILE in this study was merely due to unexplained poor results in the latter regimen and not a significant improvement of outcome due to the former regimen. The IMEP-L-asp regimen (ifosfamide, methotrexate, etoposide, prednisolone, and L-asparaginase) was examined in 22 patients with untreated advanced-stage NK/T-cell lymphoma [75]. The ORR and CR rate were 90% and 65%. Grade 3/4 febrile neutropenia was observed in 41% of patients. With a short median follow-up of 12.8 months, the 1-year OS and PFS were 75.6% and 43.3%.

These results showed that L-asparaginase-containing regimens were efficacious for advanced-stage NK/T-cell lymphoma. SMILE is still considered to be the standard. However, for elderly individuals who might not tolerate intensive chemotherapy, regimens of lesser intensity may be considered.

### Relapsed/refractory NK/T-cell lymphoma

For relapsed or refractory NK/T-cell lymphomas that have previously been treated by anthracycline-based chemotherapy, or regimens not containing L-asparaginase, salvage therapy with SMILE or similar regimens is effective. In a retrospectively study of SMILE for relapsed/refractory patients (*N* = 44, of whom 23 were advanced-stage) who had not been exposed to L-asparaginase, the ORR and CR rate were 77 and 66% [68]. With a median follow-up of 31 months, the 5-year OS was 52.3%, and the 4-year DFS was 68.2%. Another similar L-asparaginase-containing regimen AspaMetDex (L-asparaginase, methotrexate, and dexamethasone) was examined in the treatment of 19 patients (7 of them were advanced-stage) [76]. After 3 cycles of chemotherapy, the ORR and CR rate were 77.8 and 61.1%. Grade 3/4 neutropenia developed in 44.4% of patients. With a median follow-up of 26.2 months, the estimated 2-year OS and PFS were both around 40%. In another retrospective study, 13 patients with relapsed/refractory advanced-stage disease were treated with MEDA (methotrexate, etoposide, dexamethasone, and pegylated asparaginase) [77]. After 6 cycles of chemotherapy, the ORR and CR rate were 76.9 and 61.5%. Grade 3/4 neutropenia developed in 46.2% of patients. With a median follow-up of 20 months, the 1-year OS and PFS were 69.2 and 61.5%.

For NK/T-cell lymphoma relapsing from or refractory to L-asparaginase-based therapy, the prognosis is dismal. In a retrospective study, 20 patients with relapsed/refractory NK/T-cell lymphoma were treated with gemcitabine or its combination with other agents (such as dexamethasone and cisplatinum) [78]. Fourteen patients were refractory to L-asparaginase-based chemotherapy (SMILE, *N* = 11). After gemcitabine-based therapy, the ORR and CR rate were 40 and 20%. The median OS and PFS were 4.9 and 2.3 months. For patients achieving CR or PR, the PFS was 7.3 months only, reflecting the short duration of response. NK/T-cell lymphomas express CD30 in about 40% of cases [79]. In cases that expressed CD30, the anti-CD30 antibody brentuximab vedotin had been shown in anecdotal reports to be effective after failure of L-asparaginase regimens [80, 81].

### Immunotherapy in relapsed/refractory NK/T-cell lymphomas

A recent study holds much promise in improving the bleak outlook of these patients. NK/T-cell lymphoma cells express programmed death protein ligand 1 (PDL1), ligand of the inhibitory receptor PD1 on effector T-cells [82, 83]. PDL1 expression has been shown to be associated with EBV infection in lymphoma cells. In NK/T-cell lymphomas, the EBV latent membrane protein 1 upregulates PDL1 expression through the MAPK/NF-κB pathway [84]. Ligation of PD1 on effector T-cells with PDL1 on lymphoma cells leads to inhibition of T-cell activity, providing a potential mechanism for NK/T-cell lymphoma cells to evade immunosurveillance [82]. Accordingly, in this study, seven patients with relapsed NK/T-cell lymphoma failing previous L-asparaginase regimens (*N* = 7) and allogeneic haematopoietic stem cell transplantation (HSCT) (*N* = 2) were treated with the anti-PD1 antibody pembrolizumab [85]. After a median of 7 cycles of pembrolizumab, the ORR was 100%. Five patients achieved CR that was sustained after a median follow-up of 6 (2–10) months. One post-allogeneic HSCT patient developed grade 2 acute skin graft-versus-host disease, which responded to corticosteroid treatment. There were no treatment-related adverse events observed in other patients. If confirmed with larger number of patients, PD1 blockade appears to date the most efficacious treatment for relapsed/refractory NK/T-cell lymphomas.

### Autologous HSCT

The benefit of autologous HSCT as a consolidation therapy for NK/T-cell lymphomas at first CR was examined in a retrospective study of 62 patients (advanced-stage, *N* = 31) [86]. All patients were relatively young with a median age of 45.5 years. L-asparaginase-containing chemotherapy was given in 82.3% of patients and radiotherapy in 43.5% (77.4% for early-stage disease). Prior to HSCT, CR was achieved in 61.3% (71% for early-stage and 51.6% for advanced-stage) and partial remission in 38.7% of patients. After autologous HSCT, CR rate was increased to 78.3% (90.3% for early-stage and 65.5% for advanced-stage). Treatment-related mortality was 1.6%. With a median follow-up of 43.3 months, the 3-year OS and PFS were 60 and 52.4%. For early-stage disease, the 3-year OS and PFS were 67.6 and 64.5%, and for advanced-stage disease, they were 52.3 and 40.1%. These results are not different from those obtained from chemotherapy and radiotherapy. Hence, there is no indication that autologous HSCT as a first-line strategy is beneficial.

For relapsed and refractory NK/T-cell lymphoma, the exact role of autologous HSCT is undefined [87]. Patients who fail to respond to salvage therapy have very poor outcome after autologous HSCT and therefore do not derive any benefit from the procedure. Patients who achieve complete remission after salvage with L-asparaginase regimens appear to do as well as patients undergoing autologous HSCT. Hence, the role of autologous HSCT in NK/T-cell lymphoma, if any, is likely to be restricted to specific sub-populations that need to be defined by prospective studies.

### Allogeneic HSCT

Allogeneic HSCT is a potentially curative treatment for high-risk lymphoma patients, owing to its associated graft-versus-lymphoma effect. A retrospective study analyzed the results of myeloablative (*N* = 17) and reduced intensity conditioning (RIC) (*N* = 5) allogeneic HSCT for NK/T-cell lymphomas [88]. Grade 3/4 acute graft-versus-host disease (GVHD) occurred in 14.3% of patients. The overall treatment-related mortality for myeloablative and RIC HSCT was 30 and 20%. After a median follow-up of 34 months, the 2-year OS and PFS were 40 and 34%.

In the era of L-asparaginase-containing regimen, results of allogeneic HSCT appeared to be better. In a retrospective analysis of 18 patients undergoing allogeneic HSCT, 14 patients were treated with SMILE prior to transplantation [89]. Thirteen patients had advanced-stage disease at presentation, and 16 patients were at CR prior to HSCT (CR1, *N* = 9; CR2, *N* = 7). Only four patients received RIC. Grade 3/4 acute GVHD occurred in 11% of patients and chronic GVHD in 5%. Transplant-related mortality was 22%. With a median follow-up of 20.5 months, the estimated 5-year PFS and OS were 51 and 57%. The use of SMILE and achieving CR prior to HSCT were associated with a better outcome. Survivals of patients transplanted at CR1 and CR2 did not show significant differences, suggesting that allogeneic HSCT for patients in CR1 may not be justified. Given the high treatment-related mortality, allogeneic HSCT should be reserved for high-risk patients with advanced and relapsed/refractory disease. Prospective study including risk stratification is required to identify patients who may benefit from allogeneic HSCT.

### Treatment of aggressive NK-cell leukaemia

The optimal treatment of this rare disease is undefined. In an earlier study, only 3 of 13 patients treated with anthracycline-containing regimens achieved CR, resulting in a median survival of merely 58 days [90]. In another retrospective study of 18 patients treated with first-line L-asparaginase-containing regimens (SMILE, *N* = 13), the ORR and CR were 50 and 27.8% [91]. Eight patients then underwent allogeneic HSCT, with six patients remaining alive. The benefit of L-asparaginase-containing chemotherapy followed by allogeneic HSCT was also demonstrated in another study of 21 patients [92]. Seventeen patients received an L-asparaginase-containing regimen, and 14 were in CR prior to HSCT. Seven patients received RIC. With a median follow-up of 25 months, the 2-year PFS and OS were 20 and 24%. CR status at the time of HSCT was associated with significantly superior survivals.

### Prognostication of NK/T-cell lymphoma

With such a vast array of treatment options available for NK/T-cell lymphoma, prognostication models are necessary in order to triage patients to the best risk-adapted treatment. When anthracycline-containing regimens were used, the International Prognostic Index [93] and the Korean Prognostic Index [94] had been shown to stratify successfully patients into different risk categories. However, with the abandonment of anthracycline-containing regimens and the advent of non-anthracycline and L-asparaginase-containing regimens, it has become important to develop a validated prognostic model. An international consortium has recently formulated two scoring systems, PINK (prognostic index for NK/T-cell lymphoma) and PINK-E (PINK EBV) [95]. Factors found to be significant included age >60 years, stage III/IV disease, distant lymph-node involvement, non-nasal tumours on presentation (for PINK), and quantifiable circulating EBV DNA (for PINK-E). The PINK/PINK-E model will need to be prospectively validated.

Prognostic models based on presentation parameters have serious drawbacks, one of which is that the response of the lymphoma to treatment is not taken into consideration. In a cohort of SMILE-treated patients, it had been shown that at interim (after 1–2 cycles of treatment), undetectable as compared with detectable circulating EBV DNA predicted significantly superior survivals [46]. Similarly, in SMILE-treated patients, interim PET/CT (performed after 2–3 cycles) showed that normal (Deauville score ≤ 3) as compared with abnormal (Deauville score ≥ 4) findings predicted significantly superior survivals [56]. These findings show that it is possible to have a dynamic assessment of prognosis based on interim assessment results, which reflect tumour sensitivity to treatment. At end-of-treatment assessment, it had also been shown that both normal PET/CT scan (Deauville score ≤ 3) and non-quantifiable circulating EBV DNA were required to give the best survivals [48].

The prognostication of NK/T-cell lymphoma depends as much on presentation as interim and end-of-treatment parameters. Therefore, patients should be carefully evaluated throughout the treatment, in order to enable timely therapeutic modifications to be adopted.

## Conclusions

Giant strides have been made in the last two decades in our understanding and treatment of NK/T-cell lymphomas. With non-anthracycline and L-asparaginase-containing regimens, up to 90% of good-risk stage I/II patients may achieve durable remission. The treatment of high-risk stage I/II and stage III/IV patients remains challenging. The recent observation of the high efficacy of PD1 blockade suggests that immunotherapy has a major role to play in this EBV-infected lymphoma, where EBV antigens may be targets for effector T-cells. Furthermore, advances in genetic analysis will provide new leads in targeted therapy. These new developments will no doubt significantly improve the outcome of high-risk and advanced-stage patients.

## Abbreviations

CAEBV: Chronic active EBV infection
CCRT: Concurrent chemoradiotherapy
CR: Complete response
EBV: Epstein-Barr virus
FDG: Fluorodeoxyglucose
HSCT: Haematopoietic stem cell transplantation
MRI: Magnetic resonance imaging
NK: Natural killer
ORR: Overall response rate
OS: Overall survival
PET/CT: Positron emission tomography/computed tomography
PFS: Progression-free survival
TCR: T-cell receptor
TIA1: T-cell intracellular antigen 1
